# WSES position paper on vascular emergency surgery

**DOI:** 10.1186/s13017-015-0037-2

**Published:** 2015-10-22

**Authors:** Bruno Monteiro T. Pereira, Osvaldo Chiara, Fabio Ramponi, Dieter G. Weber, Stefania Cimbanassi, Belinda De Simone, Korana Musicki, Guilherme Vieira Meirelles, Fausto Catena, Luca Ansaloni, Federico Coccolini, Massimo Sartelli, Salomone Di Saverio, Cino Bendinelli, Gustavo Pereira Fraga

**Affiliations:** Division of Trauma Surgery, Department of Surgery, School of Medical Sciences, University of Campinas (Unicamp), Campinas, SP Brazil; Trauma Team, Ospedale Niguarda Milano, Milan, Italy; Department of Cardiothoracic Surgery, John Hunter Hospital, Newcastle, NSW Australia; Department of Traumatology, John Hunter Hospital, Newcastle, NSW Australia; Department of Emergency and Trauma Surgery of the University Hospital of Parma, Parma, Italy; Department of general and emergency surgery, Papa Giovanni XIII Hospital, Bergamo, Italy; Department of surgery, Macerata Hospital, Macerata, Italy; Department of surgery, Maggiore Hospital of Bologna, Bologna, Italy

**Keywords:** Trauma, Vascular injuries, Vascular control, Ruptured abdominal aorta aneurism, Vascular Trauma: Neck, Chest and Extremities

## Abstract

Trauma, both blunt and penetrating, is extremely common worldwide, as trauma to major vessels. The management of these patients requires specialized surgical skills and techniques of the trauma surgeon. Furthermore few other surgical emergencies require immediate diagnosis and treatment like a ruptured abdominal aortic aneurysm (rAAA). Mortality of patients with a rAAA reaches 85 %, with more than half dying before reaching the hospital. These are acute events demanding immediate intervention to save life and limb and precluding any attempt at transfer or referral.

It is the purpose of this position paper to discuss neck, chest, extremities and abdominal trauma, bringing to light recent evidence based data as well as expert opinions; besides, in this paper we present a review of the recent literature on rAAA and we discuss the rationale for transfer to referral center, the role of preoperative imaging and the pros and cons of Endoluminal repair of rAAA (REVAR) versus Open Repair (OR).

## Introduction

Trauma, both blunt and penetrating, is extremely common worldwide. As a result, trauma to major vessels is a not uncommon clinical occurrence. These are acute events demanding immediate intervention to save life and limb and precluding any attempt at transfer or referral. Therefore, the particular specialized surgical skills, techniques and materials for the care of these patients need to be at the disposal of the trauma surgeon. It is the purpose of this position paper to discuss neck, chest and extremities trauma, bringing to light recent evidence based data as well as expert opinions. Also, this will focus on the treatment of injured arteries, although attention will be given to those venous injuries, which require surgical repair rather than simple ligation.

The literature is filled of epidemiological researches demonstrating the features of vascular trauma in a variety of countries [1–9]. There is wide variation in the incidence, cause and mechanism of injury depending on geographic conditions. In Australia vascular injuries represent 1–2 % of total trauma patients [5–9], however it account for 20 % of all trauma related death [5]. Deaths from vascular injury diverge considerably with anatomic location and mechanism of injury. Thoracic vascular injuries routinely have death rates between 30–50 %; vascular injuries to extremities are significantly lower in the range of 5 %, in a civilian reality. In an unparalleled large study from Vietnam, Rich and colleagues [10] reported a total death of only 1.7 % for all vascular injuries. It may be that life-threatening vascular injuries were pre-selected by their failure to survive transport. In the current warfare conditions of the American intervention in Iraq and Afghanistan,Fox and his group reported that vascular trauma represents 7 % of total battle injuries, 88 % of these were extremity injuries [11]. The amputation rate was only 8 % after vascular repair. In North India [3], with a low risk of personal violence, blunt injuries, mostly motor vehicle accidents, account for 84 % of vascular injuries. Whereas in Medellin, Colombia [6] 93 % of vascular injuries are penetrating and in Georgia, USA they represent 85 % of the total [2]. Surprisingly, in the western European experience [8], up to 40 % of vascular injuries are iatrogenic, as a result of vascular and other surgical interventions. Jaha et al, recently reported a survival rate of plus 95 % for multiple mechanisms of vascular injuries in Kosovo, most of those were penetrating peripheral vascular trauma (78.3 %) [12]. Kuwait [1] strikes a middle ground with 41 % penetrating, 23 % a result of road traffic accidents (RTA). In Malaysia [4], over 50 % of vascular injuries occur as a result of RTAs differently from a British Trauma Center experience [13]. As far as anatomic site of injury is concerned, variability is less. In Australia [5–9] injuries are split almost equally between thorax, abdomen and upper and lower extremities, with cervical injuries being less common. In Latin America [7], extremity injuries are twice as common as thoracic and abdominal, although these later result in a higher mortality [14, 15]. As far as extremities are concerned upper and lower injuries occur with similar frequency and the brachial, femoral and popliteal arteries are the most commonly injured vessels [16].

## General principles for vascular injuries

Before specifically talk about neck, chest and extremities vascular injuries some basic principles must be reviewed.

Traumatic vascular lesions in general have a similar pattern of injury. They are either penetrating or blunt. Still, can be subdivided into high/ low velocity penetrating injury (i.e.: war caliber rifle injury, hand gun injury, shotgun injury, stab wound); blunt vascular injuries caused by joint displacements, bone fractures, contusions; blast injuries provoked by mines, improvised explosion devices, bombs, shrapnel, etc.

In one way or another, the pattern of injury is straight related to the kinetic energy and stretching force, ending up in general, in a similar injury-type such as contusion, total/partial transection and arterio-venous fistulae. Actual management though, may vary depending on the mechanism of injury.

The watershed here is to determine whether or not the presenting patient has palpable pulses. Clinical examination is paramount in these vascular trauma situations and the presence of distal palpable pulse (when possible to measure), even if diminished, already suggests that proximal artery injury is limited. Serial clinical examinations are mandatory. Use of a hand held bedside Doppler is extremely helpful. Table 1 presents hard/soft signs for arterial injuries, which are important to determine medical treatment.

**Table 1:** Hard signs and soft signs of arterial injury [17–19]

Hard signs of arterial injury	Soft signs of arterial injury
(Requires immediate surgery)	(Consider further examination)
External arterial bleeding	History of arterial bleeding at the scene
Rapidly expanding hematoma	Proximity of penetrating/blunt trauma to major artery
Palpable thrill	Diminished unilateral distal pulse
Audible bruit	Small nonpulsatile hematoma
Obvious arterial occlusion (6 p ‘s: pulseless, pallor, paresthesia, paralysis, poikilothermia)	Neurologic deficit
	Abnormal Ankle-Brachial pressure index (<0.9)
	Abnormal flow-velocity waveform on Doppler ultrasound

### Key management principles on vascular injuries

The care of a trauma victim begins with initial assessment and resuscitation according to the ABC Principles. These do not vary for those trauma patients with vascular injury.

Standard exposures for major arteries and veins are well defined and should be adopted in regular trauma cases. Specific surgical techniques must be mastered if successful vascular repair is to be achieved. These include: proximal and distal exposure for control with vascular clamps and loops; dissection and isolation of injured vessels including veins; heparinization (local and/or systemic); use of vascular sutures; magnification loops; assessment of injury: debridement, contusion, intimal flap and distal dissection and thombosis; selective use of temporary shunting (Argyle); anatomic repairs: with vein patch, end/end anastomosis without tension and reversed autologous vein graft for larger defects; technical details of spatulated ends, running versus interrupted sutures; distal thrombectomy; completion arteriography; fasciotomy and soft tissue coverage. Proper handling of the autogenous vein graft is important.

Attention is needed for peripheral vascular trauma in general when compartment syndrome is a complication risk factor. Suspect of compartment syndrome if prolonged period of shock, arterial occlusion, combined arteriovenous injury, need for arterial or venous ligation, crush injuries, massive tissue damage and swelling. In such cases, fasciotomy is mandatory [20, 21].

#### Key principles: author’s recommendations

- Gain proximal and distal vascular control before attempting to explore a hematoma.
- Avoid large vessels dissections when not necessary, but value good injury exposure.
- Get aware about patient’s total trauma burden and physiology.
- Decide for vascular damage control early in time (Argyle shunt).
- Balloon catheters (i.e.: Fogarty) proximal and distal to artery repair, before shunt insertion.
- Regional Heparin (50 units/ml) for arterial injuries (proximal and distal to repair). Analyze if systemic heparinization is possible.
- Completion arteriography, if patient is stable.
- Venous repair is not a must.
- Perform fasciotomy when indicated.

## Neck vascular trauma

### Blunt neck vascular injuries

Blunt neck trauma (BNT) is known to be rare occurring about 5 % of time of all neck traumas. There are various sources of blunt neck trauma and each is associated with a specific pattern of injury.

Vascular injury occurs in 1–3 % of all BNT and is associated with 20–30 % mortality. It mostly occurs with motor vehicle collisions. Rapid deceleration causes hyperflexion, hyperextension, and rotation of the neck. As a result, the vascular structures are stretched over the cervical spine leading to shearing forces on the vessels and subsequent intimal tears in the vessel wall. Hard signs and soft signs of injury must be detected. Often blunt vascular injury initially manifests in the form of acute ischemic stroke and can be delayed in onset [22]. Classic presentation includes a neurologically intact patient who develops hemiparesis after a high-speed motor vehicle crash. Evaluation by four-vessel angiography remains the gold standard given its sensitivity of 99 % but it is invasive and has a significant complication rate. CT Angiogram (CTA) has excellent accuracy in detecting clinically significant injuries [23, 24]. These modalities can be used as adjuncts to evaluation but are not first-line. Duplex Ultrasound has sensitivity of 90–95 % but it is operator dependent. In general, surgical repair is preferred over ligation and primary repair is preferred over grafting [25].

### Penetrating neck vascular injuries

Penetrating neck wounds are often dramatic and require immediate action. When the penetrating mechanism transects the platysma, it is not unclear whether patients without obvious vascular or visceral cervical injuries should not undergo routine exploration: selective exploration is the standard of care [23]. Penetrating injuries to the neck have been divided into three zones: zone 1, from the sternal notch to cricoid cartilage; zone 2, from the cricoid cartilage to the angle of the mandible; zone 3, from the angle of the mandible to the skull base.

Suspected vascular injury in zones 1 and 3 in the presence of hard signs of intra-cranial dysfunction mandates arteriography prior to exploration. Zone 2 injuries are recommended to undergo prompt exploration. Study of the neurologic outcomes in neck injuries shows that the risk of cerebral infarction is unpredictable but that repair of injured vessels gives a more favorable outcome than ligation [27].

**Fig. 1:**
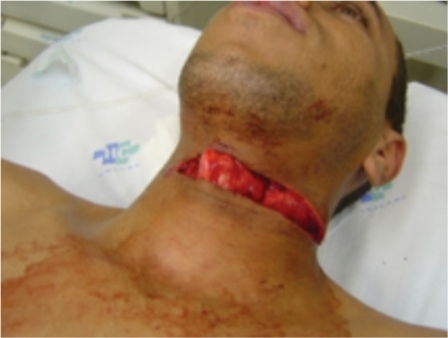
Glass-coated kite line zone II neck injury

Penetrating injuries to the neck also can vary on its mechanism. Glass-Coated kite lines – Fig. 1 [26], Stab wounds, GSW, etc. Gunshot wounds are challenging injuries to repair, are usually related to severe vascular injuries, pharynx, airways, GI, thorax. Help of an interventional radiologist for an endovascular approach is frequently required. Injuries to the vertebral artery can be tricky and very difficult to approach, especially in its zone III topography just before becoming the named basilar artery. Endovascular obliteration of the vertebral artery (VA) as well as its ligation may be preferred and a plausible solution in an *extremis* situation, although exposure of the VA isn’t easy and you may not have fast access to the angiographic suite for an endovascular procedure. In such cases, pushing a piece of bone wax into the bleeding hole usually works fine and stops the brisk bleeding coming from the VA. Internal Jugular Vein may also be ligated as a damage control measure.

For the carotid artery (CA) injuries we suggest you to go simple with no cool repairs or exotic maneuvers. Occasions affording simple repairs or end-to-end anastomosis are rare. To our experience they are usually present in low energy clean lacerations such as stab wounds caused by a dagger for instance. Our recommendation to these low complex injuries is primary repair, the use of a synthetic graft or patch to reconstruct the carotid depending on how large is the defect, when patient’s physiology allows you to proceed with this. When patient is about to breach the physiologic envelope or when there is multiple life threatening injuries associated, ligation of the CA is a valid option. When considering ligation you may be deciding for saving the patient’s life with the risk of a neurologic deficit, such as a stroke. When breaking through a zone III CA ligation is your only realistic option [28–30].

#### Authors recommendations

- Evaluate the neck fully. A C-Spine collar might obscure wounds. Take off the dressings.
- Value the mechanism of injury in blunt neck injuries.
- Be aware of insidious vascular injuries in BNT.
- Clinical signs can be similar to a stroke in BNT.
- Evaluate the location of the wounds to get a sense of the neck zone.
- If the wound does not penetrate the platysma, then it is likely that no further evaluation is required and the patient can be discharged home. If platysmal penetration is not certain, then a CT scan can be performed to rule out penetration.
- A CXR should be obtained in all circumstances (assuming the platysma has been penetrated).
- External hemorrhage should be managed by direct pressure. *Do not* probe/explore the wound. Insertion of an NG tube should be withheld until the patient is in the operating room.
- Patients should be evaluated specifically with a history and physical evaluating for changes in phonation, odynophagia, cranial nerve abnormalities, paresthesia or weakness in the extremities. *Horner*’*s syndrome* (miosis, ptosis, anhydrosis) is often missed as are physical evidence of injuries to the hypoglossal or spinal accessory nerve. If intact, document the normal exam.
- Exploration requires evaluation of the carotid sheath and its structures, the esophagus, and the larynx/trachea. The trajectory of the impaling instrument/missile should be followed. Areas might be omitted from exploration if they are not in the trajectory.

## Chest vascular trauma

### Blunt thoracic vascular injuries

Blunt thoracic aortic injury is the most severe thoracic vascular injury. This is a specific clinical syndrome related to high impact deceleration injuries and high injury severity scores. Although treatable, it causes significant mortality related partially to delayed diagnosis [25]. About 50 % of victims die on impact, in the rest the bleeding is temporarily contained by the aortic adventitia and pleura and these patients are potentially salvageable. The injury typically occurs at the site of the ligamentum arteriosum, just distal to the take off of the left subclavian artery. Shear forces and stretching of the aorta are likely mechanisms of injury. The classic sign of widened mediastinum is unreliable and investigation should be carried out in cases where there is a high index of suspicion [31]. No controversy remains regarding arch arteriography or CT scanning: Moore et al. demonstrated CT had essentially 100 % negative predictive value [31]. It appears that the use of heparin-bonded shunts allows improved results with a lower incidence of paraplegia. Endovascular techniques are playing an expanding role in the treatment of this problem [32]. Massive hemothorax requiring thoracotomy is defined as plus to 1–1.5 L at the time of insertion of chest drains or 200–300 ml/hr for the subsequent 4 h. Some of these cases will involve injury to the pulmonary vessels [33, 34]. Operative repair of aortic arch is through a median sternotomy and may require use of total cardiopulmonary bypass and insertion of a graft.

The overall incidence of blunt aortic injury has remained the same over the past 12 years despite advances in vehicle restraint systems [33].

Similar mechanisms are implicated in the injury of the non-aortic great vessels as well. Regardless of the mechanism or mechanisms, the result is vessel wall disruption, occlusion, or avulsion. Shearing can result in all of these and compression more often results in occlusion. A small intimal disruption can lead to thrombus formation and subsequent vascular occlusion [34]. Innominate artery and left carotid injuries usually occur proximally at the vessel origin. In contrast, blunt subclavian injuries tend to be more distal [11, 35].

Comparing those patients with penetrating injury, blunt thoracic great vessel injuries are less incident. In general, penetrating injuries result in higher mortality, more combined arterial and venous injures, and lower morbidity than those presenting with blunt trauma. Mortality for blunt injury has been reported between zero and 24 %.

### Penetrating thoracic vascular injuries

In thoracic penetrating injuries, the trajectory of the projectile or of the blunt object is the key to determine the anatomic structures involved. In general, missile trajectories that pass through the midline are at more risk for significant vascular injuries [36, 37].

Penetrating injuries involving the ascending arch of the aorta are uncommon. Survival rates approach 50 % for patients having stable vital signs on arrival at a trauma center. Although primary repair of anterior lacerations can be accomplished without adjuncts, cardiopulmonary bypass may be required if there is an additional posterior injury.

For an injury to the transverse aortic arch, extension of the median sternotomy to the neck allows complete exposure of the arch and brachiocephalic branches. If necessary, exposure can be enhanced further by division of the innominate vein. Simple lacerations may be repaired by lateral aortorrhaphy. With difficult lesions such as posterior lacerations or those with concomitant pulmonary artery injuries, cardiopulmonary bypass can be employed [38].

Patients with thoracic vascular injuries are either exsanguinating or have a potential bleeding contained injury. In one way or another one should be followed up in a TICU or SICU.

Bellow you find author’s recommendations for both blunt and penetrating mechanism of injury.

#### Authors recommendations by vessel injury

##### Innominate Artery& Descending Thoracic Aorta

- The proximal innominate artery and aortic arch are best approached by a median sternotomy. Early ligation of the innominate vein as well as associated thymic tissue in the anterior mediastinum will aid in exposing the aortic arch.
- The proximal descending aorta is approached by a postero-lateral thoracothomy.
- Traumatic blunt ruptures of the aorta are typically found just distal to the ligamentum arteriosum.
- For selected patients with only partial tears of the aortic arch, a continuous lateral arteriorrhaphy using 4–0 polypropylene suture is occasionally possible.
- If patients have stable thoracic hematomas and concomitant abdominal injuries for which they are unstable, laparotomy should be the initial procedure.
- For patients with rapidly expanding mediastinal hematoma, however, repair of thoracic injuries should be the primary therapeutic goal.
- Injury to the descending thoracic aorta is approached by way of a postero-lateral thoracotomy through the fourth intercostal space.
- The current standard technique of repair involves vascular clamping and direct reconstruction. Three commonly employed adjuncts to this approach include pharmacologic agents; temporary, passive bypass shunts; and pump–assisted left heart bypass.
- Vascular clamps are applied to the aortic arch, distal aorta, and left subclavian artery. Close communication between the anesthesiologist and surgeon should occur to maintain stability of hemodynamic parameters. The hematoma is entered, and care is taken to avoid indiscriminate ligation of intercostal vessels; only those required for adequate repair of the aorta should be ligated. The proximal and distal ends of the aorta are completely transected and dissected away from the esophagus. The injury then is repaired by either end–to–end anastomosis or graft interposition.
- The authors have advocated simple clamp-and-repair for injuries to the descending thoracic aorta (without the use of systemic anticoagulation or shunts), a technique that continues to be used with excellent results.
- Regardless of the technique used, paraplegia occurs in approximately 8 % of patients undergoing to descending thoracic aorta repair. Unless operative time is <30 min, partial left heart bypass is superior to clamp-and -sew in preventing paraplegia.

##### Subclavian vessels

- For subclavian injuries, a cervical extension of a median sternotomy is employed for exposure of right–sided subclavian injuries. For left subclavian artery injures, proximal control is obtained through an anterolateral thoracotomy (third intercostal space), while a separate supraclavicular incision provides distal control.
- In subclavian vascular trauma, a high associated rate of brachial plexus injury is seen.
- Documentation of preoperative neurologic status is important, in all thoracic and neck vascular injuries.
- Repair of subclavian arteries can usually be accomplished with either lateral arteriorrhaphy or graft interposition. Any difficulty in exposure can be managed with division or resection of the clavicle exposing the more distal subclavian.
- Subclavian reconstruction commonly requires the use of a graft (Dacron or PTFE). In the patient, in extremis flow can be reestablished with the use of a shunt, or the artery can be ligated as a life–saving measure.
- Operative exposure of the subclavian veins is equivalent of that described for subclavian artery injuries: median sternotomy with cervical extension for right-sided injuries and left anterolateral thoracotomy with a separate supraclavicular incision for left-sided injuries.
- Repair should be performed by either lateral venorraphy or ligation.

##### Pulmonary artery & veins

- The intrapericardial pulmonary arteries should be approached via median sternotomy.
- Exposure of the intrapericardial right pulmonary artery is achieved by dissecting between the superior vena cava and the ascending aorta.
- Mortality rates for injury to the central pulmonary arteries or veins are high (>70 %).
- When there is a major hilar injury, rapid pneumonectomy may be a lifesaving maneuver.
- Injuries to the pulmonary veins are difficult to manage through an anterior incision.
- With major bleeding, temporary occlusion of the entire hilum may be necessary.
- If a pulmonary vein must be ligated, the appropriate lobe will need to be resected.

## Extremities vascular trauma

### Upper extremity

Injuries to the upper extremity vessels are common, usually penetrating and may be associated with significant nerve and orthopedic injury. Blunt injury is usually a result of supracondylar fracture of the humerus or dislocation of the elbow. The amputation rate for ligation of the common brachial vessel varies from 18 to 55 %. With isolated injury to the infra-brachial vessels the amputation rate is lower and ligation of either the radial or ulnar arteries alone is usually well tolerated. A higher level of technical skill is required in dealing with smaller vessels and use of magnification loops is recommended. Spasm of the vessels is frequent and may require topical lidocaine or intra-arterial papaverine. Generally, prosthetic materials are not recommended. Passing of Fogarty catheters proximally and distally is important to remove eventual thrombus. Completion angiograms are important to detect abnormalities, which might result in post-operative thrombosis of the repair. Soft tissue coverage of the repair uses adjacent muscle. Fasciotomy needs to be done if ischemic time is prolonged and orthopaedic stabilization should occur after vascular repair [16–18].

#### Author recommendations

- Gain rapid access to axillary vessels through the pectoralis major muscle, extending the incision from the mid-clavicle to the deltopectoral groove.
- Damage control options for axillary artery are not many: shunt insertion, fasciotomy and/or less commonly ligation.
- Gain rapid access to the brachial vessels through a incision along the groove between the biceps and triceps muscle.
- Take the median nerve as your anatomic landmark.
- Damage control options for brachial artery are ligation (well tolerated) and fasciotomy.
- Definitive care is usually using a saphena vein interposition graft harvested just above the ankle.

### Lower extremity

As for upper extremities, penetrating injuries are more often common in the lower extremities. Pre-operative angiography may not be useful in severe trauma when taken the patient to the operating room for exploration is the best option. Surgically the use of shunts may be helpful as a damage control option, however local heparinization, passing fogarty catheters and completion angiograms can also be obtained. In general, simple vessel injuries are repaired, complex injuries ligated. If grafting is required contralateral reversed saphenous vein is recommended. Fasciotomies should be indicated based on clinical grounds and performed in all cases of prolonged ischemia time, in severe limb injuries or when there are tense compartments, combined arterial and venous lesions, in the presence of motor or sensory defect or in limbs of questionable viability. In war related injuries to the extremity fasciotomies are prophylactically recommended [11]. Primary amputation should be carried out, if the superficial posterior and one other compartment shows non-viability or in cases of devastating injuries to the limb. The amputation rate varies from 16 to 20 % and may be larger in war scenario. Attention should be given to soft tissue injuries associated to vascular lesions. Infectious complications and soft tissue injury contribute to late amputation after severe lower extremity trauma [8, 10–13, 39, 40]. Prepping the contralateral leg for possible harvesting of the long saphenous vein should be remembered.

#### Femoral vessels

Seventy percent of all peripheral vascular injuries in urban trauma centers are due to femoral injuries. The superficial femoral artery is most incident [41]. The most common complication is compartment syndrome (±19 %) and deep venous thrombosis (±13 %). In an American Civilian Trauma Center the fasciotomy rate for femoral vessels varies in around 14 %. Amputation rate in these centers are low [41].

#### Authors recommendations

- If there is urgent indication go into the abdomen and control the external iliac artery in the pelvis.
- Vertical groin incision is the simplest way to gain proximal control of the femoral artery.
- Blunt dissection is recommended in devastating vascular trauma.
- The source of persistent back bleeding is frequently the deep femoral artery. This must be identified and controlled.
- Temporary shunt and ligation are plausible damage control options for femoral vessels (Shunting is a much better option for arterial injuries). Don’t hesitate in ligate the femoral vein if needed.
- Temporary shunt for common and superficial femoral arteries is an excellent damage control solution. Authors strongly recommend a pre-emptive fasciotomy in such cases.
- Interposition PTFE grafts are well tolerated.
- Always cover your arterial vascular suture with viable well-vascularized soft tissue.
- Decide (with the orthopedic surgeons) to achieve bone alignment prior to arterial repair.

##### Popliteal vessels

Blunt injuries are more often present and carry almost 3 times the risk of amputation in comparison to penetrating injuries. Popliteal vessels are challenging to approach and treat. It is the least accessible vessel in the lower extremity and the collateral flow around the knee is not sufficient to sustain viability of the lower leg if flow of the popliteal artery is interrupted. Popliteal artery traumatic injuries carry the highest limb loss rate of all extremity vascular injuries. Injuries requiring resection of more than 2 cm are not amenable to primary anastomosis. Popliteal vein injuries, which usually occur together with arterial injuries, should be repaired if possible, but never delay the surgery time in physiologically crashed patients. In the case of combined injuries intra-arterial shunts may play a particular role. Prophylactic fasciotomy is recommended in delayed injuries or those with complex soft tissue damage. In the usual case of major soft tissue trauma the decision is often one of primary amputation versus repair. The absolute indications for primary amputation in these cases are: more than 6 h of ischemic time and disruption of the posterior tibial nerve. The relative indications are: severe foot wounds, multiple trauma, injuries requiring extensive soft tissue coverage and tibial reconstruction [42, 43].

#### Authors recommendations

- Always begin a popliteal repair with a fasciotomy.
- Proximal and distal control are mandatory (as are for all other vascular traumatic injuries).
- Think about systemic heparin whether there are no potential bleeding injuries.
- The posterior edge of the femur is the key anatomical landmark for managing popliteal injuries.
- Careful with the popliteal vein and saphenous nerve is advised when dissecting the popliteal artery.
- Go simple: bypass and exclude the injured popliteal artery.
- Saphenous vein interposition grafts are recommended.

### Abdominal vascular injury

Abdominal vascular injury (AVI) is defined as a trauma of the intra- and retro-peritoneal principal arteries and veins, accounting for the 27–33 % of all vascular trauma, and for the 25 % of all the abdominal injuries. The 90–95 % of AVI occurs after penetrating trauma, with incidences of 10 % after stab wounds and 25 % after gunshot wounds, respectively [44]. Particularly, combined injuries involving arteries and veins are common after penetrating trauma and frequent for the iliac and superior mesenteric vessels because of their anatomical proximity [45].

## Pathophysiology

*Penetrating trauma* produces AVI by different mechanisms. Through-and-through perforation or lateral defects in the wall will lead to contained haematomas, which are either pulsatile and expanding (arterial) or non-pulsatile (venous). Only a small number of patients have free haemorrhage into the peritoneal cavity. Complete transections are rarely seen, probably because uncontrolled bleeding from a large-size vessel leads to exsanguination in the pre-hospital setting. On occasion, the trajectory of a missile may be in proximity of a visceral vessel and causes a thrombosis due to the disruption of the intima from the blast effect. The rarest injury related to penetrating trauma is an artero-venous fistula, usually in the upper abdomen or in the iliac area.

*Blunt trauma* may induce AVI by deceleration forces, direct anterior crushing (lap-type seatbelt) or posterior blow (direct compression) of the structures. Two different types of injury may be caused by deceleration forces. The first one is the avulsion of small branches from the major vessels (i.e intestinal branches from superior mesenteric artery). The second is the partial intimal tear with a secondary thrombosis of the lumen, or full-thickness tear with a secondary pseudoaneurysm. A direct anterior crush and posterior blow may lead to vascular damage by an intimal tear or flap with secondary thrombosis (i.e “seat-belt aorta”)[46], and disruption of a vessel (i.e superior mesenteric artery or vein at the base of mesentery). A complete wall disruption leads to a massive intraperitoneal haemorrhage, while partial disruption produces a false aneurysm [47].

## Areas of AVI

Although any vessel in the abdomen can be injured, the term *abdominal vascular injury* usually refers to injury of major vessels located in specific “geographic” zones, as listed below:

1. Zone 1: Midline retroperitoneum.
  Supramesocolic area: suprarenal abdominal aorta, celiac axis, proximal superior mesenteric artery, proximal renal artery, superior mesenteric vein (either supramesocolic or retromesocolic);
  Inframesocolic area: infrarenal abdominal aorta, infrahepatic inferior vena cava;
2. Zone 2: Upper lateral retroperitoneum (renal artery, renal vein)
3. Zone 3: Pelvic retroperitoneum (iliac artery, iliac vein)
4. Portal-retrohepatic area: (portal vein, hepatic artery, retrohepatic vena cava).

As a general rule, all haematomas in zone 1 (either supramesocolic or inframesocolic) from either penetrating or blunt trauma have to be explored. In addition, haematomas from penetrating wounds in zone 2 and 3, and in the porta hepatis need to be opened. In contrast, haematomas from blunt trauma that are located in zones 2 e 3 or in the retrohepatic area are explored only if they are pulsatile, expanding rapidly, or have already ruptured. The classification system is applied to extra- parenchymal vascular injuries, according to the Organ Injury Score (OIS) Table 2 [48]. It is important to be aware that potential visceral injuries (i.e, duodenum, colon, stomach, small bowel, etc.) may be associated to any AVI.

**Table 2:** Classification system of abdominal vascular system [17–19]

OIS grade	Description of injuries
I	Non-named superior mesenteric artery and vein, and their branches, phrenic artery/vein, lumbar artery/vein, gonadal artery/vein, ovarian artery/vein
II	Right, left, common hepatic artery, splenic artery/vein, right or left gastric arteries, gastroduodenal artery, inferior mesenteric artery/vein, primary branches of mesenteric artery/vein
III	Superior mesenteric vein trunk, renal artery/vein, iliac artery/vein, hypogastric artery/vein, vena cava, infrarenal
IV	Superior mesenteric artery trunk, celiac axis proper, vena cava suprarenal and infrahepatic, aorta, infrarenal.
V	Portal vein, extra-parenchymal hepatic vein, vena cava retrohepatic or suprahepatic, aorta suprarenal, subdiaphragmatic

## Diagnosis

Upon physical examination, the findings in patients with AVI, after either blunt or penetrating injury mechanisms, depend on whether a *contained haematoma* or *uncontrolled haemorrhage* is present. Patients with a contained haematoma, particularly those with venous injuries, may be hypotensive, but will respond rapidly to infusions. In this case contrast CT scan is the diagnostic tool of choice. Other patients with uncontrolled haemorrhage are hypotensive and non-responsive to fluid infusion, with a tight abdomen, due to intra-peritoneal active bleeding from the damaged vessel. Abdominal distension in association with signs of acute anemia and/or haemorrhagic shock is a strong indicator of a major AVI. Another important physical finding is the loss of the pulse in the femoral artery in one of the lower extremity when the ipsilateral common or external iliac artery has been transected or is thrombosed. Rarely, the patient may be hypotensive, with a copious emesis of dark blood. In this case, a caval-duodenal fistula is suspected. The presence of a wide pulse pressure, abdominal bruit and haematuria suggest an acute aorto-caval fistula. In patients arriving with a blunt or penetrating abdominal trauma with profound hypotension or peritonitis and positive ultrasound (FAST-Focused Abdominal Sonography for Trauma), a time limit of less than 5 min in the emergency room before surgery is mandatory and CT scan is not recommended.

## Operative strategy

In the operating theatre, the patient lays in the supine position, with both arms fully abducted. The operative field extends from chin to above the knees and between the posterior axillary lines, in order to provide free access to the abdomen, chest wall and both groins. If the patient arrives profoundly hypotensive or experiences cardiopulmonary arrest in the operating room, an immediate anterolateral thoracotomy with aortic cross-clamping is performed prior to entering the abdomen. Differently, if the patient arrives with some degree of haemodynamic stability, but deteriorates during laparotomy, the abdominal aorta can be controlled digitally at the hiatus through the lesser sac or by cross-clamping. A wide vertical midline incision is carried from the xyphoid to the pubis. All clots are removed and a rapid inspection is performed to visualize a contained haematoma or an ongoing haemorrhage. Active bleeding from a solid organ is controlled by packing, while formal proximal and distal vascular control is essential for an active haemorrhage from major intraabdominal vessels. Once haemorrhage has been controlled, any eventual gastrointestinal spillage is addressed, to avoid further contamination during vascular repair. Conversely, if a contained haematoma is present, occasionally the surgeon has time to control the gastrointestinal contamination first, and subsequently to open the retroperitoneum exposing the underlying vascular injury.

## Management of injuries in zone 1: supramesocolic region

### Exposure and vascular control

The approach to an injured vessel in the supramesocolic area is different in the presence of a confined haematoma or an active bleeding:

- in the case of a confined haematoma, the vascular control is achieved by a left-sided medial visceral rotation, including the colon, kidney, spleen, tail of the pancreas and the fundus of the stomach. One alternative is to leave the left kidney in its fossa, thereby eliminating the potential damage resulting from rotation of this organ. The transection of the left crus of the diaphragm at 2 o’ clock position allows the exposure of the distal thoracic aorta and a vascular clamp is applied to obtain supraceliac aortic proximal control.
- in the presence of an active haemorrhage a manual compression may be performed. Alternatively, the lesser omentum is entered manually, the stomach and esophagus are retracted to the left, and the fibers of the aortic hiatus divided manually to obtain a quicker proximal control. Distal control of the aorta in this location is challenging because of the presence of celiac axis and superior mesenteric artery. If the ligation and division of the celiac axis are required, the surgeon must be aware of the potential gallbladder necrosis, as likely consequence. Cholecystectomy is warranted, although it may be performed during re-exploration, if damage control techniques are required. Another possible approach is an extended Kocher manoeuvre, by moving the duodenum and head of the pancreas to the left, in order to expose the suprarenal abdominal aorta.

### Vascular repair

#### Suprarenal aorta (SA)

Small perforating wounds at the SA are repaired by 3–0 or 4–0 polypropylene lateral sutures. If two small perforations are adjacent to one another, they should be connected and the defect closed transversally. If the closure of the perforations results in a significant narrowing of the lumen, or in the presence of a large defect, a patch aortoplasty with polytetrafluoroethylene (PTFE) is required. Occasionally, patients with extensive injuries require insertion of a synthetic vascular conduit or vascular graft, after resection of the involved area [49]. It is important to remember that suprarenal cross-clamping in presence of a haemorrhagic shock induces a severe lower extremities ischemia, with a subsequent reperfusion injury, once the haemodynamic stability has been restored. Compartment pressure of the legs needs to be measured before moving the patient from the operating room, and if this is higher than 30–35 mmHg, two-incision with four-compartment fasciotomies are recommended.

#### Celiac axis (CA)

Injuries of the branches of the CA are difficult to repair because of the dense neural and lymphatic surrounding tissue, and the small size of these vessels in a patient in shock with secondary vasoconstriction. If the left gastric artery and splenic artery are injured, these vessels should be ligated. Splenectomy must be performed if the splenic artery has been ligated. The larger diameter of the hepatic artery sometimes allows a lateral arteriorraphy, end-to-end anastomosis or the insertion of a graft. However, the surgeon has the option to ligate the vessel proximal to the origin of the gastroduodenal artery, since the collateral flows from the midgut through this artery will maintain the viability of the liver.

#### Superior mesenteric artery (SMA)

**Fig. 2:**
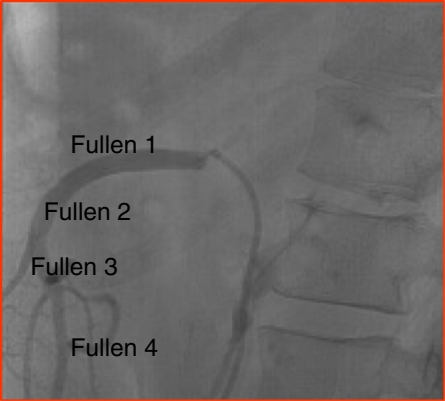
Zones of superior mesenteric artery: angiographic view was excluded

**Fig. 3:**
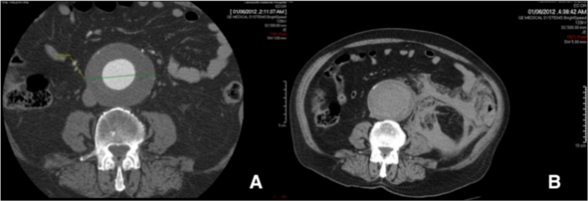
**a** Abdominal CT with IV contrast of a patient with symptomatic AAA. **b** Non-contrast abdominal CT of the same patient after collapse about 2 h later. The AAA ruptured as is evident from the retroperitoneal hematoma

The SMA is anatomically divided into four zones (Fullen zones) (Figs. 2 and 3), and the management of any injury of this vessel depends on the level of injury itself. If the injury is located behind or beneath the pancreas (Fullen zone 1 and zone 2, respectively), the transection of the pancreas, or a left-side medial visceral rotation, or elevation of transverse mesocolon, allow a direct clamping of the proximal SMA. Under all of these conditions, the artery may be ligated, and theoretically, the flow from both the foregut and the hindgut maintains the viability of the midgut (through the middle colic artery). In patients in shock and vasoconstricted collateral flow may be ineffective and the insertion of a temporary intraluminal shunt into the debrided ends of SMA is a better choice, with definitive repair during a second-look procedure. Injuries to the distal SMA (Fullen zone 3, beyond the middle colic branch, and zone 4, at the level of the enteric branches) should be repaired to avoid intestinal ischemia. If this cannot be accomplished, ligation of the artery requires an extensive resection of the ileum and right colon.

#### Superior mesenteric vein (SMV)

A part of the SMV is retro-pancreatic and difficult to expose, and has abundant collaterals. It may be approached through a Kocher and Cattell Braasch maneuver and repaired with a continuous row of 5–0 polypropylene sutures. If multiple vascular and visceral injuries are present, in a young patient, ligation of the SMV can be performed. A vigorous fluid resuscitation is needed, as a splancnic hypervolemia leads to peripheral hypovolemia for at least 3 days after ligation.

## Management of injuries in zone 1: inframesocolic region

### Exposure and vascular control

To expose an inframesocolic injury of the aorta or caval vein (IVC) the transverse mesocolon is pulled upward, small bowel eviscerated toward the right side, and the midline incised from the left renal vein to the origin of iliac vessels. In case of a large retroperitoneal haematoma, it should be remembered that the hole in the aorta is usually under the highest point of the haematoma (Mount Everest Phenomenon). A rapid finger splitting brings the surgeon to the injured area. Distal vascular control is achieved by dividing the retroperitoneum downward until aortic bifurcation. An injury involving IVC has to be suspected if the haematoma is more extensive to the right side. In this case, IVC control is obtained by a right-sided medial visceral rotation (Kocher and Cattell Braasch). The two areas in which the proximal and distal control of IVC are difficult to obtain are at the confluence of common iliac veins and at the junction of the renal veins. In the first setting it is possible to divide the right common iliac artery allowing the exposure of the iliac vein bifurcation. The artery will be reconstituted by an end-to-end anastomosis. In the second case, the medial rotation of the right kidney permits the application of a clamp. Another technique, which is useful for controlling bleeding from IVC in all locations, is the trans-femoral insertion of a Foley catheter, for tamponade.

### Vascular repair

#### Infrarenal aorta

Injuries of the infrarenal aorta are repaired primarily with 3–0 or 4–0 polypropylene sutures, patch aortoplasty, end-to-end anastomosis, or graft. In the young patient, when a graft is used or an extensive repair has been performed, it is better to cover the suture line by a vascularized pedicle of omentum, in order to prevent an aorto-duodenal fistula.

#### Infrahepatic IVC

Anterior injuries of the IVC are best repaired transversally with a running suture of 4–0 or 5–0 polypropylene. If a through-and-through perforation is present, the posterior defect is repaired first, from inside the vessel, with the first knot tied outside the lumen. If a long longitudinal suture is performed, the caval vein will appear as an hourglass, and the narrowing will lead to a postoperative occlusion of the vessel. If the patient is unstable, a modification of the repair should not be attempted. In complex injuries of the young patient, the IVC may be ligated [50]. In the postoperative course, the compartment pressure of the legs needs to be measured, and four-compartments fasciotomies must be done if the pressure exceeds 30–35 mmHg. An adequate circulating volume has to be maintained, and elastic compression applied to both lower extremities. Ligation of the suprarenal vena cava is performed only if the patient has an extensive injury at this location and appears to be in profound shock at the end of the operation.

## Management of injuries in zone 2

### Exposure and vascular control

Patients found to have a perirenal haematoma following a penetrating trauma should undergo the haematoma exploration. If the haematoma is not rapidly expanding, proximal vascular control is obtained, before entering the haematoma, by looping the ipsilateral renal vessels. Proximal renal arteries (RA) are better approached through the base of the mesocolon, beneath the left renal vein. Conversely, if there is an active bleeding, the exposure of the proximal part of the left RA is accomplished by a left-sided medial visceral rotation and of the right RA by a Kocher maneuver. The retroperitoneum is opened lateral to the injured organ, the kidney is manually lifted upward and a vascular clamp is applied proximal to the hilum. If a non-expanding haematoma is detected after blunt trauma, surgical exploration should not be attempted and post-operative angiography with interventional radiology repair should be planned.

### Vascular repair

#### Renal arteries (RA)

Small perforations from penetrating injury can be repaired by lateral sutures or resection with an end-to-end anastomosis. In the presence of large defects, graft interposition should be considered only if there is a reasonable possibility to save the kidney. After a blunt trauma, a patient with an injury to one kidney should be considered for revascularization only in the presence of stable haemodynamics and short time of ischemia (less than 5 h).

#### Renal veins (RV)

A lateral venorrhaphy is the preferred technique for repair. If right RV has to be ligated, a nephrectomy should be performed at the same time or at the reoperation if damage control has been necessary. Left proximal RV can be ligated as long as the gonadal veins and the left adrenal vein are intact.

## Management of injuries in zone 3

### Exposure and vascular control

The proximal vascular control of the iliac vessels is obtained by eviscerating the small bowel to the right and opening the retroperitoneum over the aortic bifurcation. Distal vascular control is achieved at the point the vessels come out of the pelvis proximal to the inguinal ligament [51]. When bilateral injuries are present, the only way to achieve bleeding control is the total pelvic vascular exclusion, with a proximal cross-clamping of the aorta or vena cava, above their bifurcation, and a distal cross-clamping of the external iliac arteries or veins on both sides.

### Vascular repair

#### Iliac arteries (IA)

Injuries of the common IA should be sutured or temporarily shunted if possible. Ligation of these vessels in the hypotensive patient leads to limb ischemia. In a stable or stabilized patient, depending on the type of injury, it is possible to perform a lateral arteriorraphy, an end-to-end anastomosis and an insertion of saphenous vein or PTFE graft. External IA may be ligated if omolateral internal IA is intact.

#### Iliac veins (IV)

Injuries of the IV are best repaired with a 4–0 or 5–0 polypropylene lateral suture or with ligation. If a significant narrowing of the lumen has occurred after the repair, postoperative anticoagulation therapy should be started to avoid thrombosis and pulmonary embolism.

## Management of injuries in the porta hepatis

### Exposure and vascular control

It is important to be aware that vascular injuries at this location are frequently associated with an injury of the common bile duct. Because of this anatomic proximity, no suture should be placed until the vascular injury is precisely defined. If haematoma or haemorrhage are present the Pringle’s maneuver (compression of the hepatoduodenal ligament between non crushing clamps, fingers or loops) should be used. The injuries of the portal vein are best exposed with a wide Cattel-Braasch maneuver. Exposure of the posterior position of the vessel requires an extensive Kocher maneuver in association with a mobilization of the common bile duct toward the left and the cystic duct superiorly. The approach to retropancreatic portion of the vessel requires pancreatic transection followed by distal pancreatectomy once the repair is made.

#### Hepatic artery (HA)

Lateral repair or shunt of HA are difficult because of its small caliber, but desirable. In fact, the portal vein alone is not always sufficient for liver viability [52]. Ligation of the gastro-hepatic artery, proximal to the origin of gastroduodenal artery is usually well tolerated. Ligation of the right HA requires a cholecystectomy.

#### Portal vein (PV)

Direct lateral venorraphy with a 4–0 or 5–0 polypropylene suture is the technique of choice. It is better to avoid any attempt to perform a porto-systemic shunt, because of the possible onset of encephalopathy at a later stage. Ligation of PV has a very high mortality. Massive fluid sequestration induces transient splancnic hypervolemia that requires a large amount of fluid restoration to avoid peripheral hypoperfusion.

## Recommendations

All trauma surgeons must be skilled in the techniques of emergency abdominal vascular control and repair. The reduced functional reserve of the unstable patient with AVI and the presence of multiple injuries require a damage control approach with staged surgical strategy. Moreover, an abdominal vascular ligation or repair in more stable patients needs to be re-explored for the assessment of visceral viability. Finally, AVI are unusual after blunt trauma, which in Europe account for 95 % of trauma admissions. Further, training for the treatment of these injuries is generally low. For all these considerations, a patient with an AVI represents one of the most challenging scenarios for a trauma team and mortality remains elevated.

### Non traumatic emergency vascular surgery

#### Ruptured aneurysms of the abdominal aorta: current management and results

Few other surgical emergencies require immediate diagnosis and treatment like a ruptured abdominal aortic aneurysm (rAAA). Mortality of patients with a rAAA reaches 85 %, with more than half dying before reaching the hospital. Open repair (OR) of rAAA is associated with perioperative mortality of 40–70 %. Patient age, haemodinamic instability and pre-existing comordibitidies are significantly associated with perioperative deaths. Endoluminal repair of rAAA (REVAR) has emerged as an alternative to OR. Since the first experience with this technique in the early 1990s, a substantial decrease in perioperative and long term mortality has been demonstrated after REVAR when compared with OR. In addition to the advances of REVAR, modern resuscitation techniques including hemostatic resuscitation and permissive hypotension, and the availability of endoluminal aortic occlusive balloons for supraceliac aortic control in the emergency room, has assisted salvaging patients that historically died before reaching surgery. REVAR mandates pre-operative imaging, a dedicated team, an angio suite and ready access to suitable stents.

In this paper we present a review of the recent literature on rAAA. We discuss the rationale for transfer to referral center, the role of preoperative imaging and the pros and cons of REVAR versus OR.

## Where should ruptured abdominal aortic aneurysms be repaired?

A positive correlation between sub-specialization, high volume and improved outcome has been demonstrated in multiple surgical procedures, including open and endovascular operations. Thus, a dilemma occurs: should an open repair be attempted immediately, by a team and center with limited experience, or should the patient be transferred to the referral centre for sub-specialized care with obvious delay to treatment and risk of decompensation during transfer? Relatively sparse data is available on the true effects of transfer of patients with an unsecured rAAA. A recent case-series in Canada suggests that while the transfer caused treatment delays (from approximately 3 to 6 h), it did not significantly impact mortality (50 vs 54 %) [53]. However, the retrospective design of this study may have biased by selection of likely survivors for transfer, and palliation of the sicker patients. Unfortunately, for vascular emergencies, the last decade has seen an unchecked drive towards elective subspecialization with a surge of endovascular procedures and a rapid reduction of open vascular procedures. In this environment, the General and Acute Care Surgeons have lost almost all training in vascular surgery. The large variations in local practices and expertise, retrieval teams, and geography preclude a universal recommendation on a transfer policy.

We suggest preemptive, careful design of pathway and protocols for patients with a rAAA which should be tailored to individual hospitals and area health. In any case, preoperative resuscitation should follow the concept of “permissive hypotension” with the aim of maintaining consciousness and prevent ST changes, and a systolic blood pressure between 70 and 80 mmHg [54, 55]. This can be achieved by limiting infusion of fluids and blood products and/or pharmacologically reducing the blood pressure [56].

## Role of preoperative imaging

**Fig. 4:**
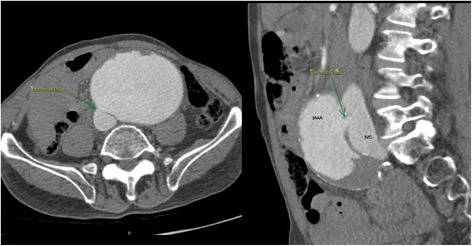
Abdominal CT with IV contrast demonstrating a large infrarenal AAA ruptured in the inferior vena cava in axial and sagittal view

**Fig. 5:**
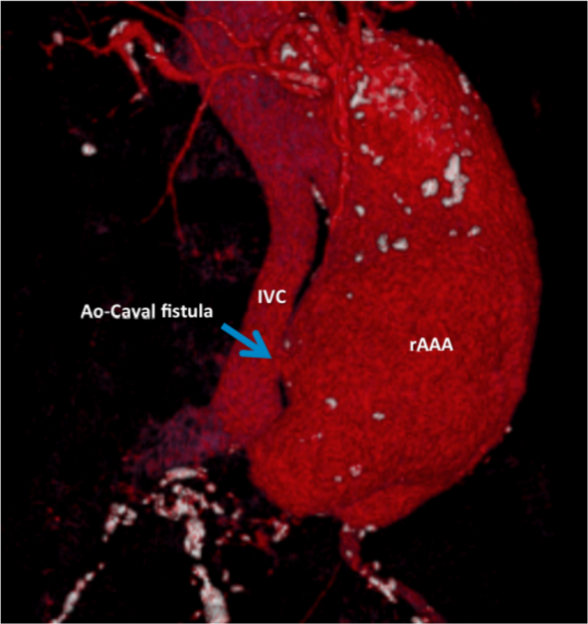
3D reconstruction of large infrarenal AAA ruptured into the inferior vena cava

While open repair of a rAAA can be “attempted” with minimal imaging by most surgeons familiar with abdominal surgery, REVAR introduced the need of pre-operative CT scan. To allow immediate and accurate imaging reconstruction, CT should be performed with intravenous contrast, by a trained radiographer, with the correct bolus timing, no oral contrast and possibly the availability of thin axial slices. A good quality CT scan is of paramount importance to properly assess feasibility of endoluminal repair and graft sizing (Fig. 3, Fig. 4, Fig. 5). The main concern of running a potentially unstable patient through a scanner is the possible delay from ED admission to surgery. According to Lloydet al.,in not operated patients with rAAA the mean time from onset of symptoms and ED admission to death was 16 and 11 h respectively; only 13 % of patients died within 2 h of admission. In case of transfer, the CT scans can be reviewed online and EVAR suitability and measurements can be completed before the patient arrives to the hospital. The lack of protocols for efficient transfer and management of patients with rAAA, rather than the time spent in radiology, seems to cause most delays to surgery [57–59].

Patients with a systolic blood pressure above 80mmHG should be preoperatively investigated with CT with IV contrast. Patients with a systolic less then 80 mmHg should be immediately transferred to theatre with an aortic occlusive balloon (endoclamp) ready to be inflated in the supra-coeliac aorta. REVAR assessment can then be done with a digital subtraction angiogram on the operative table.

## Open repair

The open approach to the rAAA has been standard for several decades. During this time, relatively little has changed regarding the surgical procedure: first, rapid proximal control of the aneurysm is achieved immediately on induction of anesthesia; second, distal control; and finally, after a small period of time is afforded to the anesthetic team to further resuscitate the patient, the aneurysm is opened and a graft used to bypass the diseased aorta [60, 61].

Preoperative CT imaging is not strictly necessary, but still very useful as it can guide in location to achieve proximal control and possible anatomical variation (such as a retroaortic renal vein or a horseshoe kidney) or presence of additional aneurysms.

The rAAA is classically approached through a midline laparotomy, facilitating a transperitoneal repair. This approach provides good exposure of the abdominal aorta and common iliac arteries. The peritoneal exploration also allows inspection of the abdominal viscera for secondary pathology. However a retroperitoneal approach may be preferable in cases such as known supraceliac extension of the aneurysm, a battle-scarred abdomen, or in patients with atypical anatomy such as a horseshoe kidney. While there are no randomized data to guide the type of incision for a rAAA, in cases of elective repair, randomized trials have produced conflicting results regarding possible reductions in postoperative ileus and shorter hospital stays associated with a retroperitoneal approach [62, 63].

After entering the peritoneal cavity, routine supraceliac control has traditionally been the first maneuver. However, when the aneurysm is limited to the infrarenal aorta, aortic control may be achieved below the renals in a similar time frame and is a feasible alternative: the assistant temporarily compresses the supraceliac aorta against the vertebral body at the diaphragm’s aortic hiatus, while the surgeon uses fingertip exploration of the periaortic hematoma to guide an infrarenal clamp. Care is required to avoid venous injuries to the left renal, gonadal and inferior mesenteric veins during infrarenal clamping, as injuries to these veins are associated with a significantly worse prognosis [60]. For supraceliac control, however, the left lobe of the liver is first mobilized and retracted to the right. The nasogastric tube then facilitates identification of the esophagus, which together with the stomach is retracted towards the left. This allows access to the aorta at its diaphragmatic hiatus, through the lesser sac. Caution is required to avoid the not infrequent presence of an aberrant left hepatic artery travelling through the lesser omentum. The aorta can then be clamped as it emerges between the crura of the diaphragm.

When the rAAA is approached from a retroperitoneal dissection, the incision is usually placed through a 10th intercostal space, though in the cases of very proximal aneurysmal disease, a formal throacoabdominal incision, with a transpleural aortic cross-clamp, may be required. Retrospective series report reduced gastrointestinal and respiratory morbidity, reduced hospital stays and possible reduced mortality, favoring a retroperitoneal approach [64, 65]. During this approach, a left medial visceral rotation facilitates the aortic exposure.

Distal control is usually easier to achieve; the level of control, and the site for the distal anastomoses, will be guided by associated vascular pathology in the iliac and femoral vessels. If a bifurcated graft is used to bypass iliac disease, attempts should be made to perfuse at least one internal iliac artery to avoid ischaemic complications of the pelvis and lower abdominal viscera.

Endoluminal balloons provide an alternative to the traditional atraumatic clamps, limited by the need for a careful dissection to avoid injury to adjacent structures. First described with Foley catheters deployed through the aneurysm and inflated in the proximal aorta, endoluminal balloons have become increasingly available and are associated with reduced intraoperative mortality [66]. Furthermore, aortic occlusion catheter kits are commercially available to facilitate blind insertion and aortic control in the emergency department [67]. However, blind application of this device is not recommended in rAAAs.

After proximal and distal control, the largely decompressed aneurysm may be opened to allow the graft repair. Red cell scavenging forms a standard part of arAAA repair. Its use has been clearly linked to improved survival and reduced post-operative complications [68, 69]. It does not replace the need to careful assessment and targeted replacement of blood components, but it does provide a feasible, cost-effective, method to replace red cell loss from the vascular space.

Once the aortic reconstruction is completed before the proximal clamp is released to establish distal flow, the distal vessels are generously back bled, to prevent embolization of soft clot that may have formed. This is particularly important if the patient is neither systemically nor peripherally heparinized. As indicated by a recent review, the pro-coagulant, inflammatory stimulus from the trauma associated with a rAAA and its surgery, likely favors routine heparin administration. However, unfortunately a limited number of studies are available to guide the decision regarding the use of heparin in rAAA. As such, there remains no clear consensus or practice guideline to delineate the standard practice [70, 71].

At the completion of the vascular repair, approximately one quarter of patients will have abdominal contents too swollen to allow a non-tensioned abdominal closure, and around half of patients will exhibit an intra-abdominal pressure > 20 mmHg. Historically, these patients had their abdomens closed under tension with deleterious consequences. The resultant abdominal compartment syndrome (ACS) is responsible of multiorgan failure and increased mortality. A proactive approach to allow early diagnosis and intervention is required. Temporary abdominal closure devices with negative pressure dressing have redefined the standard of care of these patients [72, 73].

## Endoluminal repair: protocols and technique

**Fig. 6:**
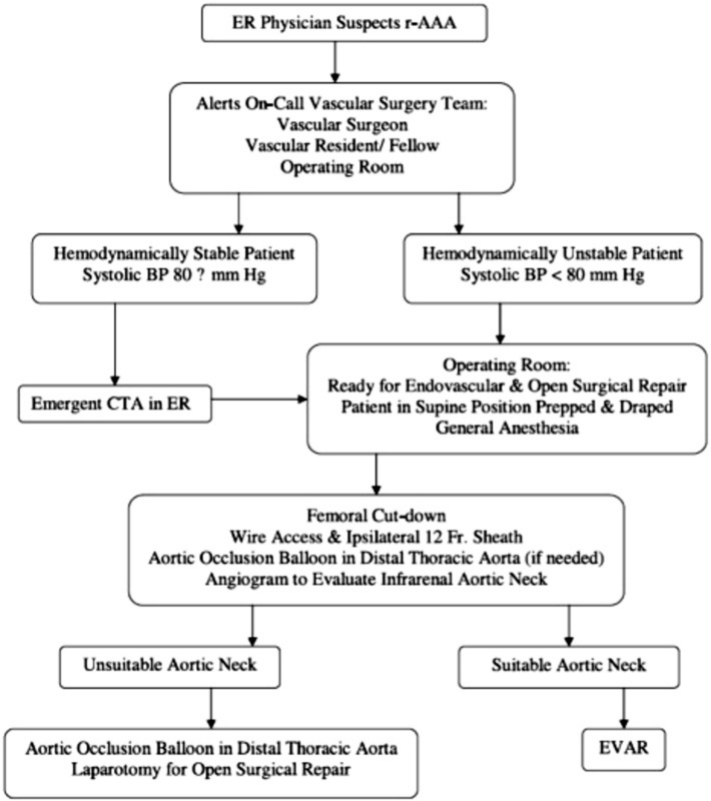
Flowchart of the rAAA protocol introduced by the Albany Group [80]

**Fig. 7:**
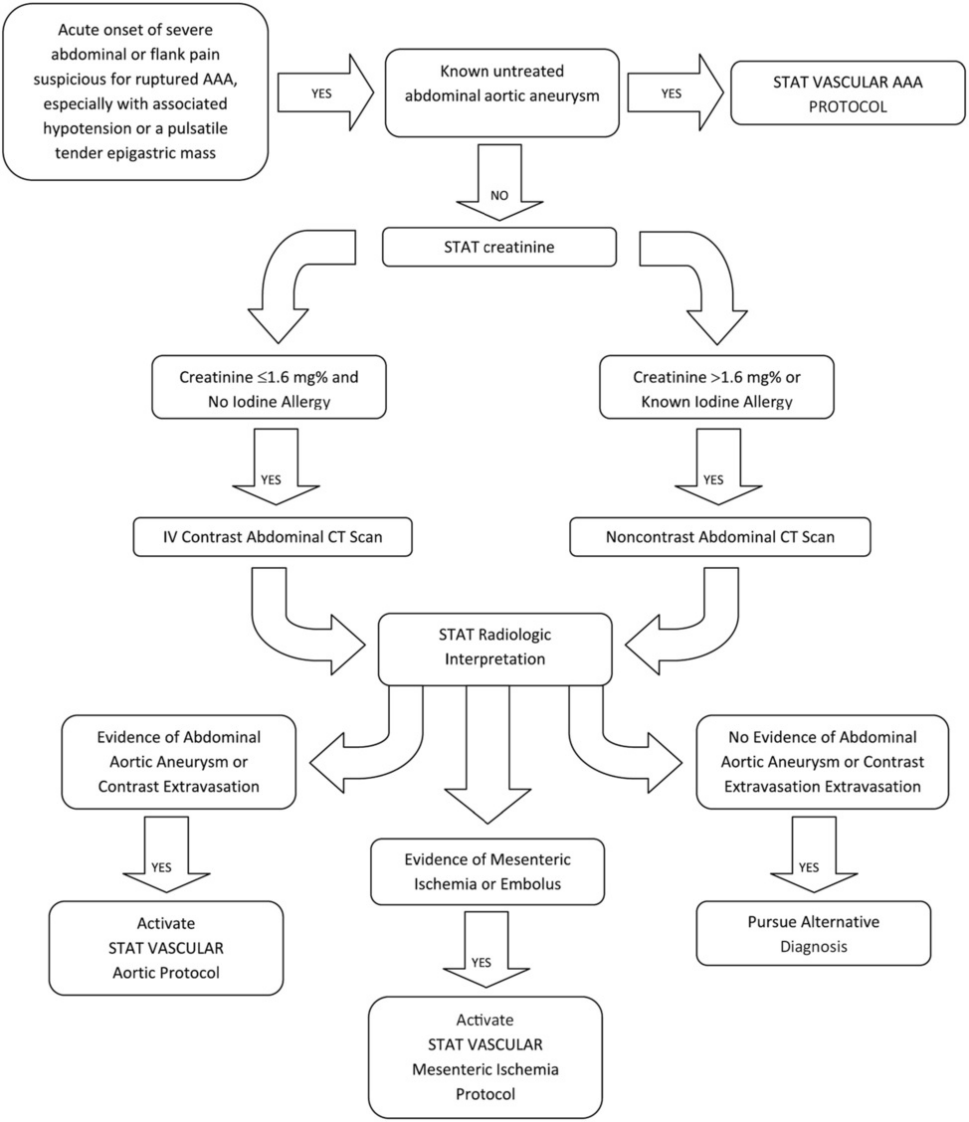
Flowchart of the STAT VASCULAR program from the University of Illinois [82]

Endovascular aneurysm repair (EVAR) is the endoluminal exclusion of an aneurysm sac from the circulation by the use of an endograft; initially described by Parodi et al. in 1991 [73], this technique has evolved and has been proven safe and effective when compared to traditional OR in the elective settings [75, 76]. In 1994 the first successful endoluminal repair of a rAAA was performed in New York [77]. Today REVAR represents the most important innovation in rAAA management over the last 50 years [78, 79]. The protocol introduced by the Albany group [80] is efficient and applicable in most tertiary centers (Fig. 6); it requires multidisciplinary teamwork and appropriate team training. Once the ED physician has a suspect of rAAA (clinical or supported with ultrasound) the vascular team and theatre staff needs to be immediately notified. A dedicated theatre fully equipped for open and endovascular surgery is mandatory; a hybrid suite is ideal, but a standard theatre with a mobile C-arm intensifier is enough. Stable patients (SBP ≥ 70–80 mmHg) will have an expeditious CT scan; unstable patients will be directly transferred to theatre for endovascular-first approach and conversion to open if necessary. Patients are usually excluded from REVAR if (i) the aortic neck length ≤ 10 mm, (ii) the aortic neck diameter ≥ 32 mm, (iii) neck angulation ≥ 75° and (iv) bilateral iliac diameter ≤ 5 mm. Using those anatomical criteria almost 80 % of patients are feasible for REVAR [80]; Mayer et al. have recently reported 100 % feasibility of REVAR over a period of 32 consecutive months, with an exclusion rate of only 4 % [81]. STAT VASCULAR is the program implemented by Hodgonsat the University of Southern Illinois (Fig. 7) [82]. It emphasizes the importance of CT angiography for all patients suspected of having an acute aortic event either abdominal or thoracic; positive CT finding activates STAT VASCULAR and the on-call vascular team. REVAR can be safely and more effectively performed under local anesthesia supplemented by analgo-sedation [83]. Femoral access is achieved either with a cut-down or percutaneously. The percutaneous approach requires familiarity with preclose techniques with ProGlide or ProStar closure devices [84]. Once vascular access is established an initial glidewire is exchanged for a stiff one over a 5Fr catheter and a 12–14Fr 45 cm long sheath is placed at the level of the renal arteries to support an aortic occlusion balloon (femoral approachis preferred over brachial) [80, 85].

In hemodynamically stable patients the balloon can be removed after the initial angiogram and the main body of the endograft will be delivered through the ipsilateral groin. In patients in hemorrhagic shock, when the balloon needs to remain inflated to maintain brain and heart perfusion, the endograft is delivered through the contralateral groin and quickly deployed after the balloon is deflated and retrieved proximally.

Different endografts are available for treatment of rAAA in both bifurcated and aorto-uniiliac (AUI) configuration and the device choice depends on the patient’s anatomy and the surgeon preference. Table 3 summarizes the characteristics of the most commonly used devices. The use of AUI devices should be limited to when (i) rapid cannulation of the contralateral gate is not possible or when (ii) the contralateral iliac is difficult to access because of occlusive disease. It is important to have a wide range of sizes and configurations available off the shelf in order to accommodate different anatomies. Adjunctive procedures (extension cuffs, giant Palmaz stents and embolization) might become necessary to seal on the table a proximal type for type IIendoleak, especially if causing ongoing hemorrhage [84, 86]. Finally extending the indications for REVAR to “all comers” might require visceral debranching (open or endoluminally with Chimney grafts) if the native neck is too short [87, 88].

**Table 3:** Characteristics of the most commonly used endografts for rAAA

	Cook Zenith®		Medtronic®	Gore®
	LP AAA	Flex AAA, Spiral-Z Iliac	Endurant II	Excluder AAA
				
Indications for use				
Infrarenal neck fixation				
Diameter	18–28 mm	18–32 mm	19–32 mm	19–32 mm
Length	≥15 mm	≥15 mm	≥10 mm or ≥15 mm	≥15 mm
Angle (to suprarenal)	<45 deg	<45 deg	≤45 deg or ≤60 deg	≤60 deg
Angle (to aneurysm)	<60 deg	<60 deg	≤60 deg or ≤75 deg	≤60 deg
Iliac (ipsi/contra) fixation				
Diameter	8–20 mm	7.5–20 mm	8–25 mm	8–25 mm
Length	>10 mm	>10 mm	≥15 mm	≥10 mm
Iliofemoral access				
Sheath inner diameter	16Fr (5.3 mm)	18–22Fr (6.0–7.3 mm)	–	18–20Fr (6.1–6.8 mm)
Sheath out diameter	18Fr (6.0 mm)	21–26Fr (7.1–8.5 mm)	18–20Fr (6.0–6.7 mm)	20–27Fr (6.8–7.5 mm)
Graft specifications				
Materials				
Stents	Nitinol (self-expanding)	Stainless steel and nitinol	Nitinol	Nitinol
Grafts	Polyester (& PTFE)	Polyester	Polyester	ePTFE (& FEP)
Modular system, bifurcated main body device				
Fixation/sealing	Suprarenal bare stent, barbs, radial force	Suprarenal bare stent, barbs, radial force	Suprarenal bare stent, barbs, radial force	Graft sealing cuff, barbs, radial force
Diameter (~15 % oversize)	22–32 mm	22–36 mm	23–36 mm	23–35 mm
Length (body-ipsilateral)	94–152 mm	112–170 mm	124–166 mm	120–180 mm
Length - body-contralateral	70–128 mm	82–140 mm	70–80 mm	70–90 mm
Modular system, iliac extension device				
Diameter (~15 % oversize)	10–24 mm	9–24 mm	10–28 mm	12–27 mm
Length (limb)	36–120 mm	39–122 mm	82–199 mm	70–140 mm
Aortouniiliac device				
Fixation/sealing	–	Suprarenal bare stent, barbs, radial force	Suprarenal bare stent, barbs, radial force	Graft sealing cuff, barbs, radial force
Diameter (~15 % oversize)	–	22–36 mm	23–36 mm	23–31 mm
Length (body-ipsilateral)	–	130–161 mm	102 mm	120–180 mm
Ancillary components				
Main body extension	45–58 mm length	39–73 mm length	49–70 mm length	33–45 mm length
Converters	66 mm length	80–82 mm length	–	–
Iliac plugs	–	14–24 mm diameter	–	–

The obvious advantage of avoiding a laparotomy carries the intrinsic risks of increased incidence of ACS. Metha reports an incidence of ACS of 18 %, occurring mainly in patients with pre-operative hemodynamic instability [89]. They suggest to routinely withhold systemic heparin in REVAR and to closely monitor the bladder pressure during and after the case. Increased bladder pressure alone or signs of end-organ dysfunction associated with abdominal distention, regardless of the bladder pressure, warrants decompressive laparotomy. Mayeret al. suggested an intravescical pressure > 20 mmHg or an abdominal perfusion pressure < 50–60 mmHg as indication for open abdominal treatment [90, 91]. Finally, on-table conversion to open surgical repair might be needed; the use of the occlusive aortic balloon as endo-clamp can be very valuable to maintain haemodinamic stability. This needs to be supported to avoid its prolapse into the AAA with consequent loss of aortic occlusion.

## Open or endovascular repair for rAAA?

Patients presenting with a ruptured aorta represent a medical and surgical challenge for everyone involved in their care. Rapid diagnosis of this catastrophic condition triggers an immediate and coordinated series of actions involving different health care providers. Despite the advances in medical care and surgical technique, the perioperative mortality rate of OR has seen only a modest improvement in the last 50 years [92, 93]. Among many theories, the “two-hit” hypothesis has been suggested to explain high mortality secondary to multi-organ failure [94]; this is summarized by the combination of two consecutive ischemic events (hemorrhagic shock and aortic clamping) followed by reperfusion injury. This sequence seems to be responsible of cardiac contractile dysfunction and massive neutrophils activation with resultant generalized peroxidation injury.

In the last twenty years multiple centers around the world have reported a dramatic reduction in perioperative mortality following REVAR [54, 74, 79, 95–100]; those excellent outcomes have been challenged as being the result of patient selection or publication biases [78, 100–103]. A meta-analysis of 18 observational studies (>10 cases) of patients undergoing REVAR; the pooled mortality among 436 pts who underwent REVAR was 21 % (95 % CI 13 to 29) but with substantial heterogeneity among different studies; however, 90 % of the heterogeneity between studies was not explained by chance alone. Surgical volume explained substantial heterogeneity [78]. A prospective study from 49 centers from all over the world showed an overall mortality at 30-days of 21.2 % but those results had an intrinsic selection bias due to the limited use of REVAR in “stable” patients in most of the centers. Thirteen centers performed “REVAR whenever possible” including haemodinamically unstable patients, and 30-days mortality was 19.7 % for REVAR compared with 36.3 % for OR (*p* < .0001) [79].

So the question is: which patient with a rAAA would benefit the most of an endograft? Do we have the data to justify the “EVAR-first” approach on everyone? Mayer et al. have reported a progressive increase in the use of REVAR over the years, reaching the up to a “100 % EVAR” approach. Adjusted 30-days mortality in the “EVAR/OPEN period” was 15.7 % for EVAR and 37.4 % for OR (*p* = 0.004). When all rAAA were treated endoluminally, 30-days mortality climbed to 24.3 % of “*all comers”* [81, 90]. Extending the indications for REVAR to “*all comers”* had multiple consequences: “exclusion from treatment” rate fell from 10 to 4 %, at the expenses of a higher REVAR mortality and more complex procedures. In addition, in order to accommodate also unsuitable anatomies, adjunctive procedures were used in 24 % of the cases, adding a considerable degree of complexity to an already challenging procedure. A Kaplan-Meier analysis based on more than 40.000 patients from the US Medicare dataset showed a survival benefit for REVAR over OR for the first 90 days; after propensity score matching the benefit persisted over 4 years [98]. Similar findings on long-term survival were also reported by Mehta et al.(37 vs 26 % REVAR and OR; *p* < .005) [95]. In this series almost a fourth of patients treated with an endograft, required re-intervention for endoleaks or graft migration, highlighting the importance of close follow-up.

A recent review based on US Medicare data beneficiaries used propensity score matching to show a survival benefit for REVAR (33.8 vs 47.7 %) which persisted at 4 years. At 36 months, EVAR patients had higher rates of AAA-related re-interventions than OR patients whereas OR patients had more laparotomy-related complications [103]. Based on a cohort of 1447 patients with rAAA, unstable patients showed less favorable outcomes: the 30-day mortality for unstable patients was 52.8 % for OR and 35.6 % for REVAR (*P* < .001),while for stable patients mortality was 26.3 % for OR and 16.4 % for REVAR (*P* = .001). Also, in this study REVAR was associated with a diminished 30-day mortality and morbidity [104]. Despite the excellent immediate and mid-term REVAR results reported by observational and population-based studies, the level of evidence to support “EVAR-first” for rAAA is still debatable [105] and all three randomized control trials conducted so far failed to prove a survival benefit with REVAR [106–110]. The first study was a single center trial conducted in the UK and published in 2006; it failed to prove a survival benefit for REVAR over OR, but results were considered inaccurate because the trial was interrupted when only a third of the predetermined number of patients was recruited.

The AJAX study [108] is a three-centers trial conducted in the Netherlands where 116 patients with rAAA anatomically suitable for “both” EVAR or OR were randomized to either treatment. This trial did not show a significant difference in combined death and severe complications between the two modalities; 30-days mortality was 21 % post REVAR compared to 25 % with OR (ARR = 4.4 %; 95 % confidence interval:−11 to +20 %). The authors explain the unusually low surgical mortality with the introduction of round-the-clock acute aneurysm service, centralization of aneurysm care and routine pre-operative CTA. This study has indeed some significant statistical and technical limitations. Only 22 % (116/520) of rAAA diagnosed in the seven years study period met anatomical criteria for randomization: this limited the yearly caseload per trial center and affected the power of the study with regard to the primary endpoints.

IMPROVE [107] is a multicenter “pragmatic” trial a total of 613 patients with rAAA randomized to REVAR or OR. REVAR was not associated with significant reduction in either 30 day mortality or cost (“endovascular strategy” 35.4 % *vs* OR 37.4 %; odds ratio 0.92; 95 % confidence interval 0.66 to 1.28; *P* = 0.62). Only potential advantages of REVAR were: (i) lower 30 day mortality in the female population (*P* = 0.02) and (ii) earlier recovery with direct discharge to home (189/201 (94 %) *vs*141/183 (77 %); *P* < 0.001 %). Despite the considerable size and the “real world” design of this study, the results from the IMPROVE trial have to be considered carefully. Among the 316 patients randomized to “*endovascular strategy”*(mortality 35.4 %; 112/316) only less than half were considered endoluminal candidate based on CTA and actually underwent REVAR (30 day mortality 25 %; 38/150). The rest were either considered anatomically unsuitable for REVAR and underwent OR (mortality 38 %; 43/112), died during conversion from REVAR to OR (100 %; 4/4), died without treatment (mortality 94 %; 16/17) or didn’t have a confirmed rAAA (mortality 33 %; 11/33); this clearly influenced the overall 30 day mortality of 35.4 % of the “*endovascular”* arm of the study. Finally strategy decision and technical expertise, with only a minimum of 5audited REVAR on the logbook, is questionable.

We conclude with a recommendation to transfer patients with clinical suspect of rAAA to centers that can offer *both treatments* with audited results.
